# Supplementary material for: Metabolomics-Driven Discovery of an Introduced Species and Two Malaysian Piper betle L. Variants
Source: Plants (Basel). 2021 Nov 19;10(11):2510. doi: 10.3390/plants10112510 (PMC8622403; doi:10.3390/plants10112510)
Supplement: Supplementary file 1 [file plants-10-02510-s001.zip › plants-1424496-supplementary.pdf]

Supplementary materials

Metabolomics-driven Discovery of an Introduced Species and Two Malaysian *Piper betle* L. Variants

Muhamad Faris Osman [1,2], Soo Yee Lee [1], Shahrul Razid Sarbini [3], Siti Munirah Mohd Faudzi [1,4], Shamsul Khamis [5], Badrul Hisyam Zainudin [6] and Khozirah Shaari [1,*]

[1] Natural Medicines and Products Research Laboratory (NaturMeds), Institute of Bioscience, Universiti Putra Malaysia, UPM, Serdang 43400, Selangor, Malaysia; farisosman@iium.edu.my (M.F.O.); daphne.leesooyee@gmail.com (S.Y.L.); sitimunirah@upm.edu.my (S.M.M.F.); khozirah@yahoo.com.my (K.S.)

[2] Department of Pharmaceutical Chemistry, Kulliyyah of Pharmacy, International Islamic University Malaysia, Kuantan 25200, Pahang, Malaysia

[3] Department of Crop Science, Faculty of Agricultural Science and Forestry, Universiti Putra Malaysia, Bintulu 97008, Sarawak, Malaysia; shahrulrazid@upm.edu.my

[4] Department of Chemistry, Faculty of Science, Universiti Putra Malaysia, UPM, Serdang 43400, Selangor, Malaysia; sitimunirah@upm.edu.my

[5] Department of Biological Sciences and Biotechnology, Faculty of Science and Technology, Universiti Kebangsaan Malaysia, UKM, Bangi 43600, Selangor, Malaysia; shamsulk@ukm.edu.my

[6] Analytical Services Laboratory, Chemistry and Technology Division, Malaysian Cocoa Board, Cocoa Innovation and Technology Centre, Lot 12621 Kawasan Perindustrian Nilai, Nilai 71800, Negeri Sembilan, Malaysia; badrul@koko.gov.my

* Correspondence: khozirah@yahoo.com.my; Tel.: +6013-3420686

Figure

**S1**	Full representative ^{1}H-NMR spectra of three "sirih"	3
**S2**	PCA score and loading plots of preprocessed ^{1}H-NMR spectral data	4
**S3**	Correlation-scaled PCA biplot for "sirih merah"	5
**S4**	Excerpt from DNA barcoding report on identification of "sirih merah"	6
**S5**	Leaf samples included in or excluded from GC-MS metabolomics	7
**S6**	Six sampling locations of *P. betle* variants and *P. rubro-venosum*	8
**S7**	Photographs of *P. betle* inflorescences	10
**S8**	BPC of *P. rubro-venosum* leaf essential oils in GC-MS analysis I	13
**S9**	BPC of *P. rubro-venosum* leaf essential oils in GC-MS analysis IIa	16
**S10**	BPC of *P. betle* leaf essential oils in GC-MS analysis I	19
**S11**	BPC of *P. betle* leaf essential oils in GC-MS analysis II	21
**S12**	Score plots when different reference files were used in MS-DIAL (GC-MS analysis I)	24
**S13**	Score plots when different reference files were used in MS-DIAL (GC-MS analysis II)	25

Table

**S1**	Description of *P. betle* and *P. rubro-venosum* studied in GC-MS metabolomics	9
**S2**	Yield percentage of *Piper* leaf essential oils	11
**S3**	Constituents of *P. rubro-venosum* leaf essential oils identified in GC-MS analysis I	12
**S4**	Constituents of *P. rubro-venosum* leaf essential oils identified in GC-MS analysis IIa	14
**S5**	Constituents of *P. betle* leaf essential oils identified in GC-MS analysis I	18
**S6**	Constituents of *P. betle* leaf essential oils identified in GC-MS analysis II	20
**S7**	Parameter settings in MS-DIAL version 4.24	26

References 27

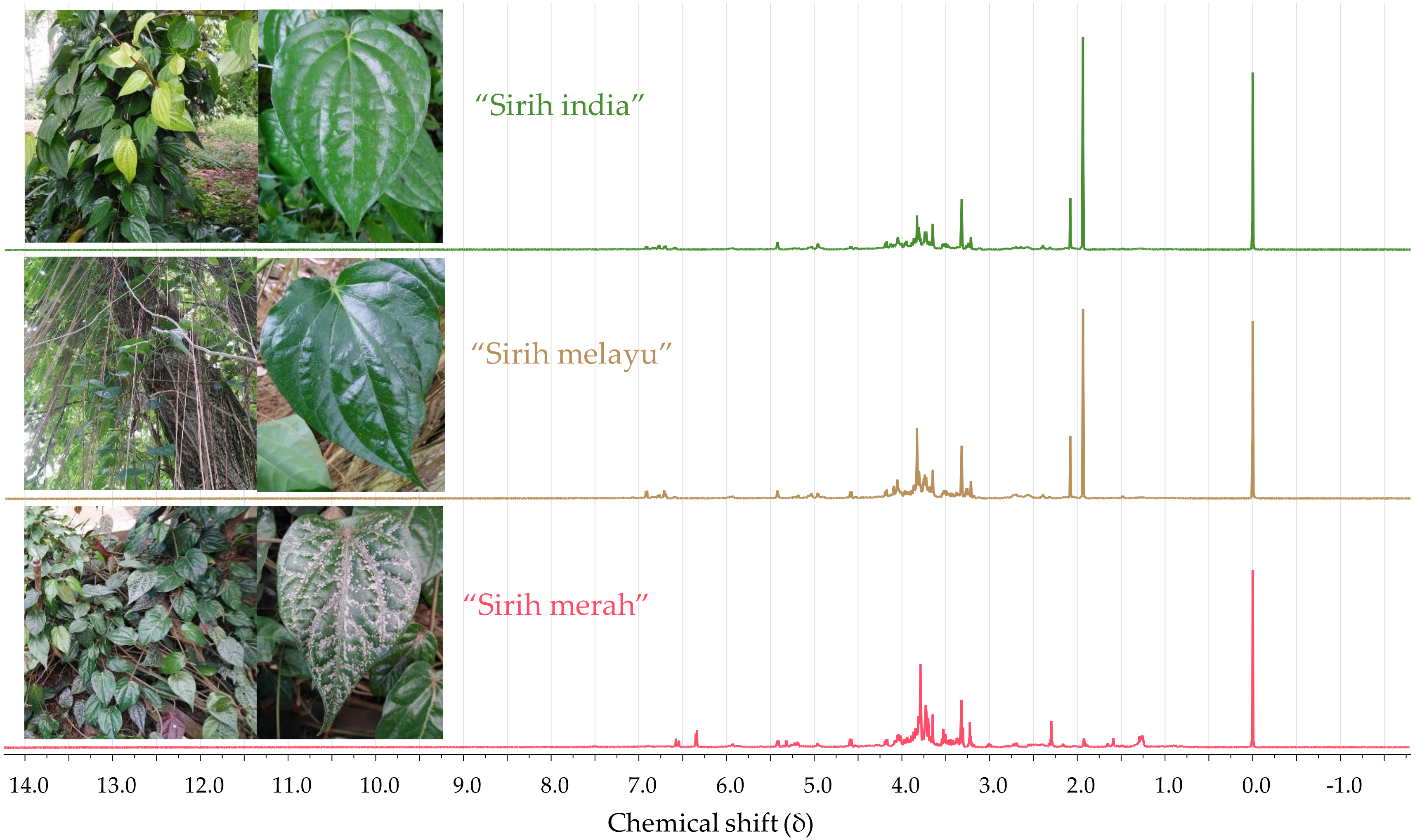

Figure S1. Full representative ^{1}H-NMR spectra of three "sirih" leaf aqueous methanolic extracts. The spectra were normalized to TSP peak at δ 0.00 (intensity of TSP peak = 100).

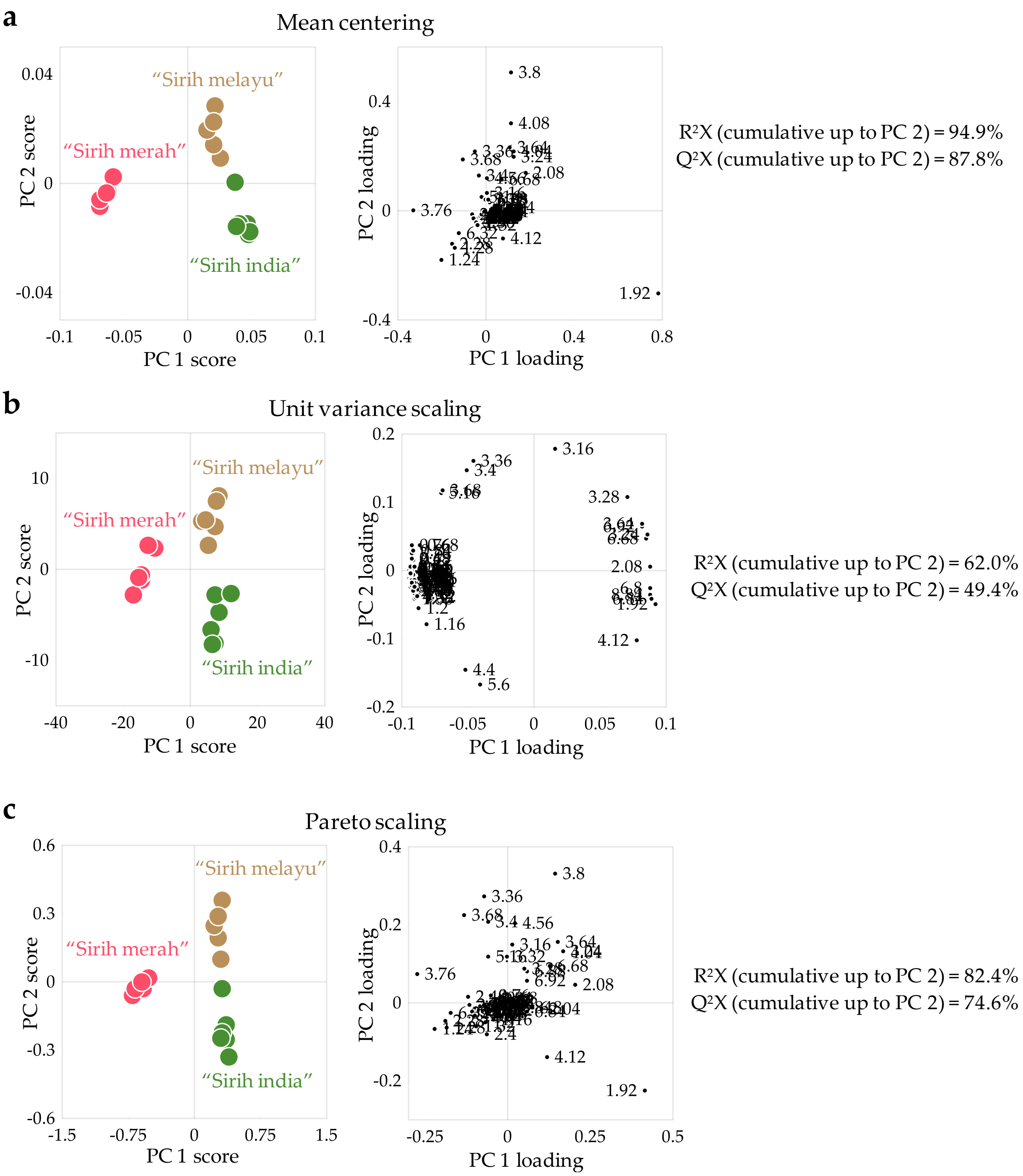

Figure S2. PCA score and loading plots of preprocessed 1H-NMR spectral data of three "sirih". Only variables that are well-modeled by the first two PC are shown in the loading plots (cumulative percentage of variation of the variable predicted by the first two principal components, as estimated by cross-validation $\geq$ 80%). R^2X = cumulative percentage of variation explained by the specified PC, Q^2X = cumulative percentage of variation predicted by the specified PC. (**a**) Score and loading plot of mean centered data matrix; (**b**) Score and loading plot of unit variance-scaled data matrix; (**c**) Score and loading plot of unit Pareto-scaled data matrix.

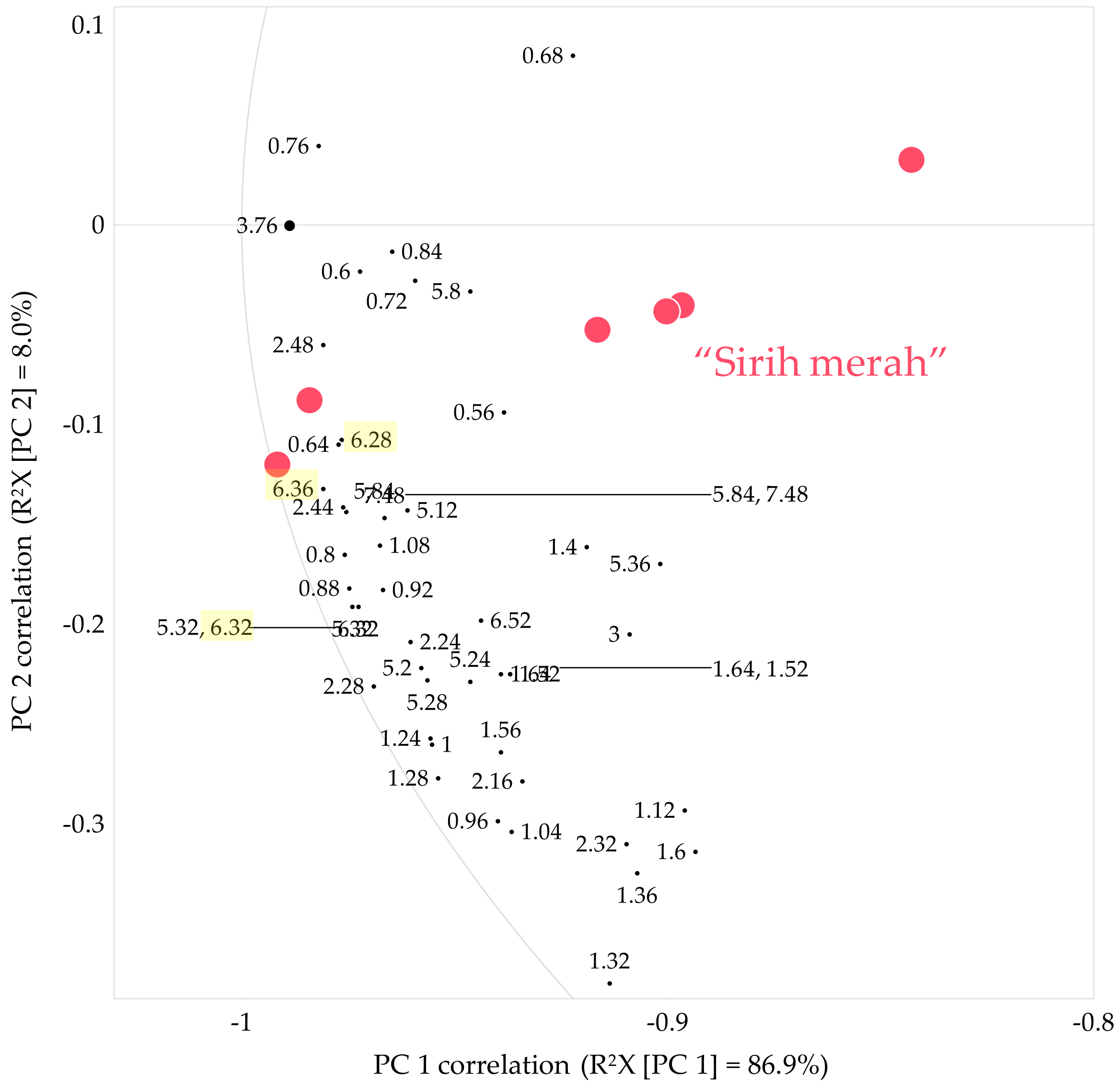

Figure S3. Correlation-scaled PCA biplot of mean-centered data matrix showing characteristic variables of "sirih merah". Variables of aromatic region are highlighted in yellow. Full view of the plot is shown in Figure 1.

FORENSIC DNA TESTING
FOR PLANT SPECIES IDENTIFICATION AND
TIMBER TRACKING

REPORT TO
MUHAMAD FARIS BIN OSMAN
(Institute of Bioscience, Universiti Putra Malaysia, 43400 UPM Serdang, Selangor)
Test Code : MGF0221
Dr. Tnah Lee Hong, Dr. Kevin Ng Kit Siong & Dr. Lee Soon Leong
Genetics Laboratory Forest Research Institute Malaysia
52109 Kepong, Selangor, Malaysia
8 April 2021

On 25th March 2021, two samples (maroon-underside leaves (RM) and green-underside leaves (RG)) claimed to be *Piper ornatum* were received from MUHAMAD FARIS BIN OSMAN, UPM for species authentication. The samples RM and RG were designated as 016F21 and 017F21, respectively.

DNA sequences of ITS2 region:

>016F21_ITS2
TTGCGCCCGAGGCCTTTCGGTCGAGGGCACATCTGCTTGGGCGTTAAACAACTCGTCGCCCACCGCCTTCTCCCT
CGCAGGGCCACGATTGTCAGCGCGTTGCGCCGATCGCTTCTCGATTGCCCAACAAGTACCCACCACAATCGAAAG
TGCGGCGCAAGCGGTTGGCTGAAAAGTTTGGGCCACGGGCTGCGTGGGGCTCAACGAGTGGTGGTTGTGCGCCCT
CCACCGCACTCCGAGCGAGGAGTGTGTTCGAGAGACGGGGGGGTAGGTTGGGCGGAGTTTGGTCGTCCGTGTGCC
GCACCCGATCCGGGGATCGATTCGAATTGCAACCCCAAGTCAGGTGGGACTACCCGCCGAGCTTAAGCATATCAA
TAAGCGKAGGAAAAGAAACTTACAAGGATTCCCCTAGTAACGGCGAGCGAACCGGGAGT
>017F21_ITS2
TTGCGCCCGAGGCCTTTCGGTCGAGGGCACATCTGCTTGGGCGTTAAACAACTCGTCGCCCACCGCCTTCTCCCT
CGCAGGGCCACGATTGTCAGCGCGTTGCGCCGATCGCTTCTCGATTGCCCAACAAGTACCCACCACAATCGAAAG
TGCGGCGCAAGCGGTTGGCTGAAAAGTTTGGGCCACGGGCTGCGTGGGGCTCAACGAGTGGTGGTTGTGCGCCCT
CCACCGCACTCCGAGCGAGGAGTGTGTTCGAGAGACGGGGGGGTAGGTTGGGCGGAGTTTGGTCGTCCGTGTGCC
GCACCCGATCCGGGGATCGATTCGAATTGCAACCCCAAGTCAGGTGGGACTACCCGCCGAGCTTAAGCATATCAA
TAAGCGKAGGAAAAGAAACTTACAAGGATTCCCCTAGTAACGGCGAGCGAACCGGGAGT

Findings and Conclusion:
Based on DNA sequences of ITS2 region, BLAST results showed 100% identity between 016F21 and 017F21. BLAST results against reference sequences from NCBI, GenBank showed 87.978% similarity with *Piper ornatum* but showed highest hit (99.387%) with *Piper rubro-venosum*.

Query ID	Subject ID	% identity	Alignment length	Misma -tches	Gap opens	Evalue	Bit score
016F21	017F21	100	434	0	0	0	781
016F21&017F21	*Piper rubro-venosum*_MH493581.1	99.387	326	2	0	1.93E-168	580
016F21&017F21	*Piper fragile*_MH493373.1	98.773	326	2	1	1.22E-164	568

Neighbor-joining analysis based on DNA sequences of ITS2 region also revealed that the 016F21 and 017F21 were clustered under one well-supported clade with *Piper rubro-venosum*.

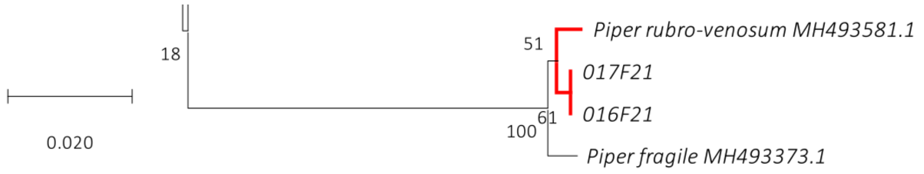

Therefore, based on these findings, we hereby confirm that 016F21 and 017F21 belong to a same species and both can be identified as *Piper rubro-venosum*.

Figure S4. Excerpt from DNA barcoding report on identification of "sirih merah" as *Piper rubro-venosum* hort. ex Rodigas.

a

b

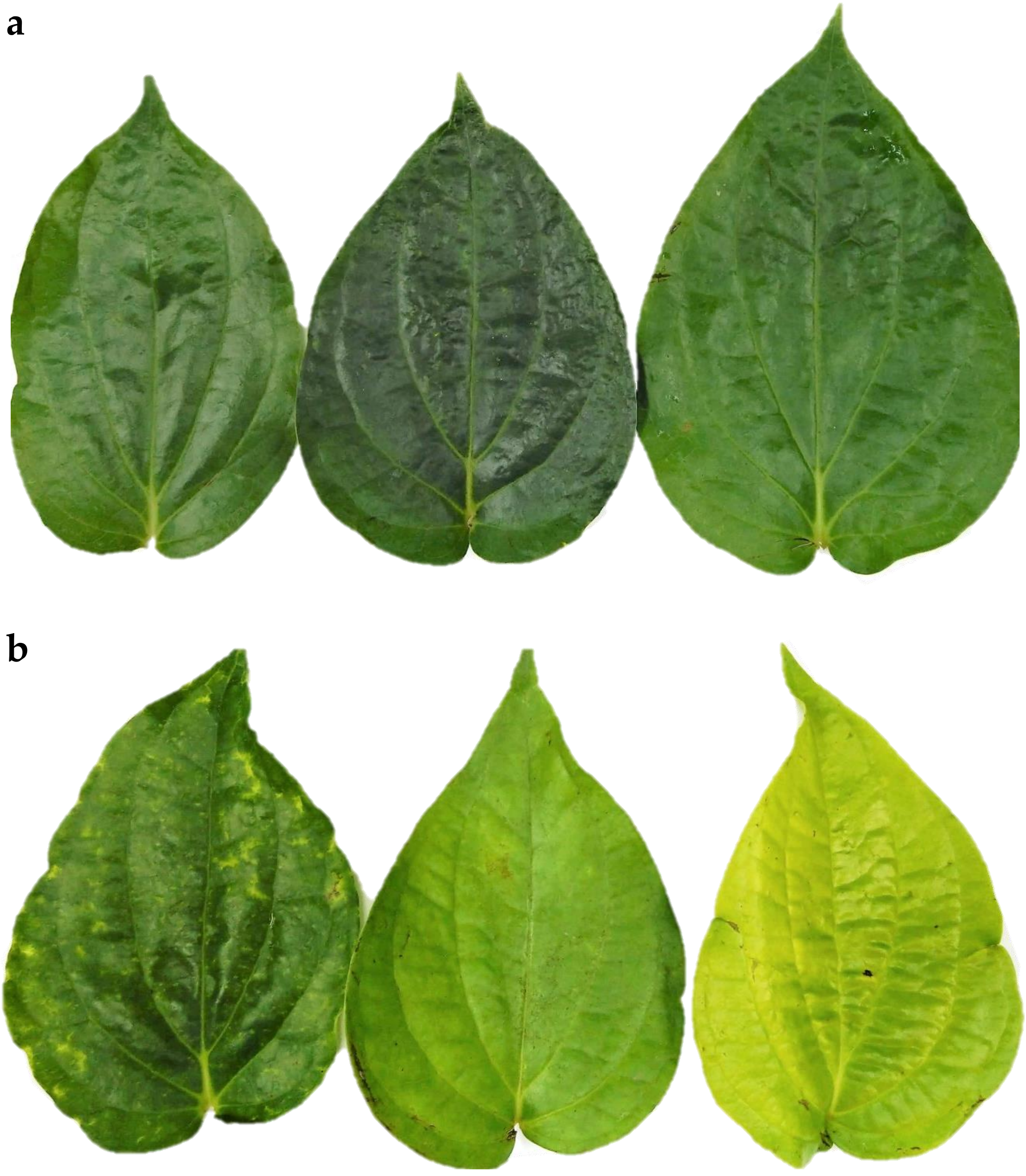

Figure S5. Leaf samples included in or excluded from GC-MS metabolomics. Example for leaves of *P. betle* "india" is shown. (**a**) Good quality leaves included in GC-MS metabolomics (healthy, unwrinkled, dark green mature leaves); (**b**) Unsatisfactory quality leaves excluded from the study (left: wrinkled leaf, middle: soft, light green young leaf, right: yellowish green leaf).

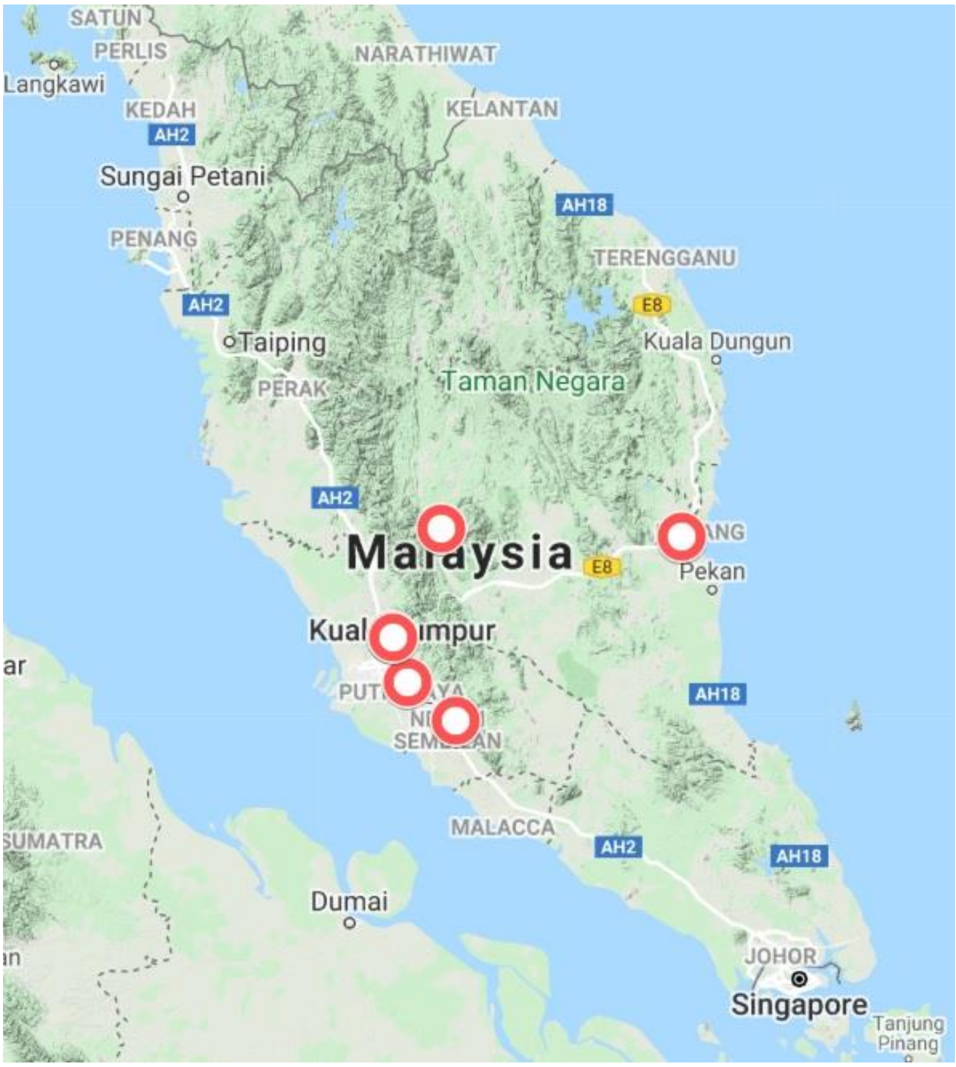

Figure S6. Six sampling locations of *Piper betle* variants and *Piper rubro-venosum* studied in GC-MS metabolomics. GPS coordinates and closer view of the six sampling locations can be viewed online at https://rb.gy/c8jnqx .

Table S1. Description of *Piper betle* variants and *Piper rubro-venosum* studied in GC-MS metabolomics.

Samples*	Collection date (Time)	Location	GPS coordinate (Altitude)	Growing condition	Collector	Occurrence of inflorescence**	Sample code
P. betle 'melayu'	19.08.2018 (13:15)	Kuantan, Pahang	3° 47' 00.6" N, 103° 13' 00.0" E (18 m)	Grown under shade of nearby trees, in front of a local resident's house. Planted for personal consumption.	Collected by Muhamad Faris Osman (M.F.O.), *n* = 5	Yes (see Figure S7)	Y1-Y5
	03.09.2018 (13:30)	Kepong, Selangor	3° 14' 11.4" N, 101° 37' 31.2" E (107 m)	Grown under shade of a big tree, next to a local resident's house. Planted for personal consumption.	Collected by M.F.O., *n* = 1	No	YA
P. betle 'india'***	22.08.2018 (16:00)	Raub, Pahang	3° 49' 45.4" N, 101° 53' 53.1" E (132 m)	Grown in rows, under shade of dark netting at a *P. betle* farm. Planted for supply to local markets.	Collected by farm's owner, *n* = 6	Yes (see Figure S7)	W1-W6
	05.09.2018 (17:00)				Collected by farm's owner, *n* = 2		WA, WB
P. betle 'manis'	29.09.2018 (11:30)	Pantai, Negeri Sembilan	2° 46' 28.6" N, 101° 58' 29.9" E (98 m)	Growing in rows, under shade of dark netting at a *P. betle* farm. Planted for supply to local markets.	Collected by M.F.O., from six rows of plants	No	M1-M6
P. betle (unknown variant)	03.09.2018 (14:15)	Kepong, Selangor	3° 13' 45.8" N, 101° 38' 14.7" E (81 m)	Grown next to a big tree, behind a local resident's house. Planted for personal consumption.	Collected by M.F.O., *n* = 1	No	XA
P. rubro-venosum	29.08.2018 (12:09)	Serdang, Selangor	2° 59' 21.5" N, 101° 42' 30.5" E (50 m)	Grown under shade of a transparent roofing system, as an ornamental plant.	Collected by M.F.O., from one plant (*n* = 1) that has leaves with green abaxial surface (RG) and maroon abaxial surface (RM)	Yes (see Figure 2)	RG
	30.08.2018 (12:11)						RM

*Variant names that are used by Malaysians to describe characteristic attributes of the *P. betle* variants. The variant name 'melayu' means Malay in English, referring to a variant that is mostly planted by Malaysians of Malay ethnicity. The variant name 'india' refers to a variant planted and consumed mostly by Malaysians of Indian ethnicity. The variant name 'manis' means sweet flavor in English, referring to a variant that has less pungent flavor.

**At time of samples collection.

***Samples collection was carried out on two different dates (2 weeks apart) to observe if there was any significant variation inflicted by the collector (farm's owner). The collector collected the samples as per specifications detailed out by M.F.O. and delivered the samples to M.F.O.

a

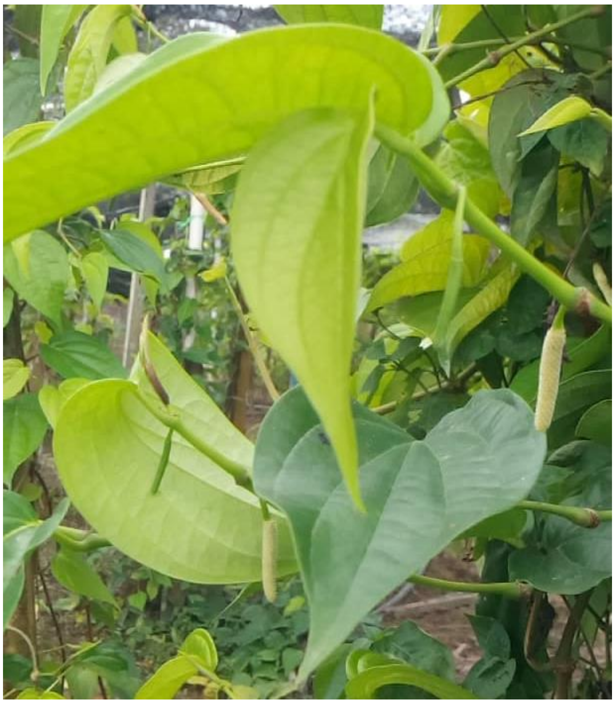

b

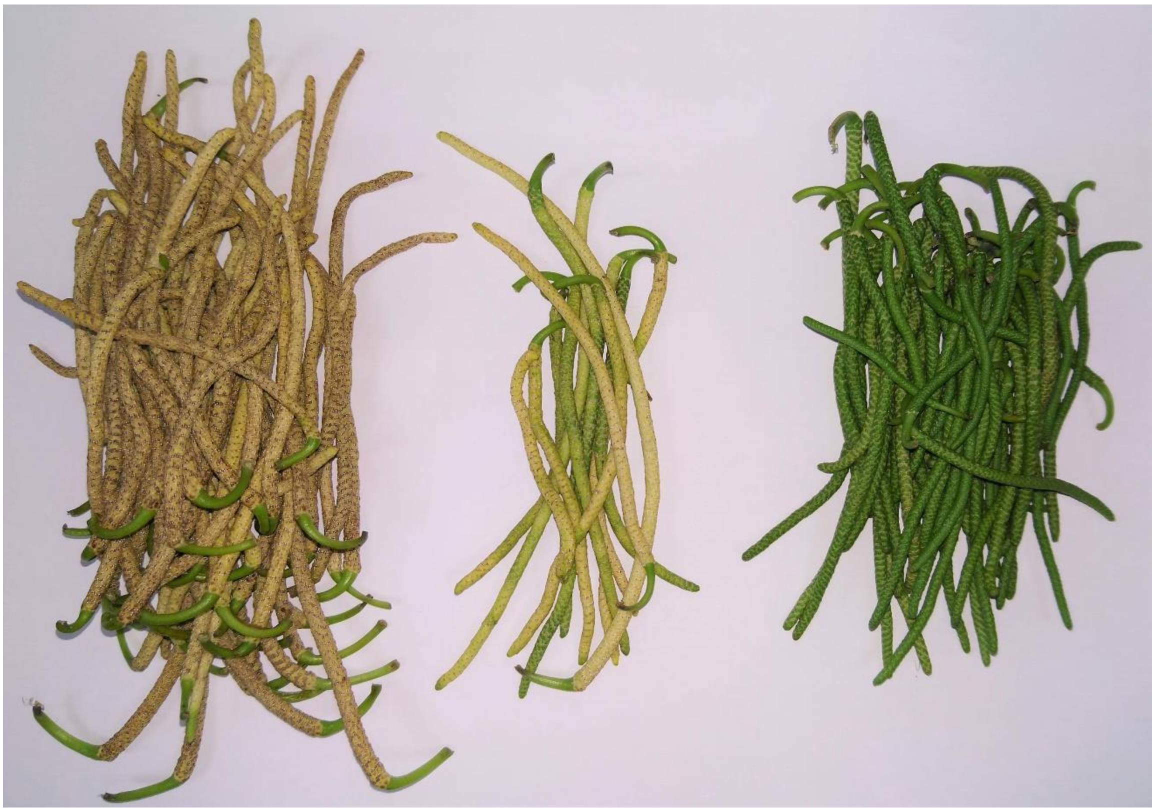

Figure S7. Photographs of *Piper betle* inflorescences (spikes) (see Table S1). (**a**) Short spikes of *P. betle* 'india'; (b) Long spikes of *P. betle* 'melayu'.

Table S2. Yield percentage of *Piper* leaf essential oils (petioles removed).

Sample code	No. of fresh leaves	Total weight of fresh leaves (g)	Average weight of fresh leaves (g)	Yield percentage of essential oil (%)
Y1	237	500.0	2.1	0.47
Y2	173	498.0	2.9	0.68
Y3	165	396.5	2.4	0.58
Y4	198	500.0	2.5	0.67
Y5	212	500.0	2.4	0.90
YA	166	398.0	2.4	0.48
W1	220	475.0	2.2	0.05
W2	220	475.0	2.2	0.07
W3	220	498.0	2.3	0.07
W4	182	388.0	2.1	0.08
W5	237	515.0	2.2	0.05
W6	110	262.0	2.4	0.05
WA	246	500.0	2.0	0.07
WB	300	661.0	2.2	0.05
M1	57	238.0	4.2	0.08
M2	95	461.0	4.9	0.14
M3	70	224.5	3.2	0.09
M4	67	249.0	3.7	0.09
M5	42	193.0	4.6	0.06
M6	92	442.0	4.8	0.11
RG	170	132.0	0.8	0.02
RM	150	276.0	1.8	0.01
XA	83	200.0	2.4	0.05

Table S3. Constituents of *Piper rubro-venosum* leaf essential oils identified in GC-MS analysis I. Constituent peaks are labelled in **Figure S8**.

No.	RT (min)	LRI	Constituent	Reference
1	9.38	973	Sabinene	[35]
2	9.95	990	Myrcene	[35]
**3**	**11.99**	**1047**	**(*E*)-β-Ocimene**	[35]
**4**	**12.41**	**1059**	**γ-Terpinene**	[35]
**5**	**12.71**	**1067**	**(*Z*)-Sabinene hydrate**	[35]
**6**	**13.51**	**1089**	**Terpinolene**	[36]
**7**	**13.98**	**1101**	**Linalool**	[35]
**8**	**16.87**	**1179**	**Terpinen-4-ol**	[35]
**9**	**17.40**	**1193**	**α-Terpineol**	[35]
**10**	**22.81**	**1338**	**δ-Elemene**	[35]
**11**	**24.47**	**1378**	**α-Copaene**	[35]
12	24.90	1388	β-Bourbonene	[35]
**13**	**25.18**	**1395**	**β-Elemene**	[35]
**14**	**26.57**	**1423**	**(*E*)-β-Caryophyllene**	[35]
**15**	**26.99**	**1431**	**β-Copaene**	[35]
**16**	**28.26**	**1455**	**α-Humulene**	[35]
**17**	**29.54**	**1479**	**γ-Muurolene**	[35]
**18**	**29.82**	**1485**	**Germacrene D**	[35]
19	30.63	1500	Bicyclogermacrene	[36]
**20**	**30.82**	**1503**	**α-Muurolene**	[36]
**21**	**31.14**	**1508**	**Germacrene A**	[36]
**22**	**31.42**	**1512**	**β-Curcumene**	[36]
**23**	**31.61**	**1515**	**γ-Cadinene**	[35]
**24**	**32.17**	**1524**	**δ-Cadinene**	[35]
**25**	**36.02**	**1583**	**Caryophyllene oxide**	[35]
26	36.76	1594	Salvial-4(14)-en-1-one	[35]
**27**	**37.82**	**1609**	**Humulene epoxide II**	[36]

Note: Constituents in bold were identified in GC-MS analysis I and IIa.

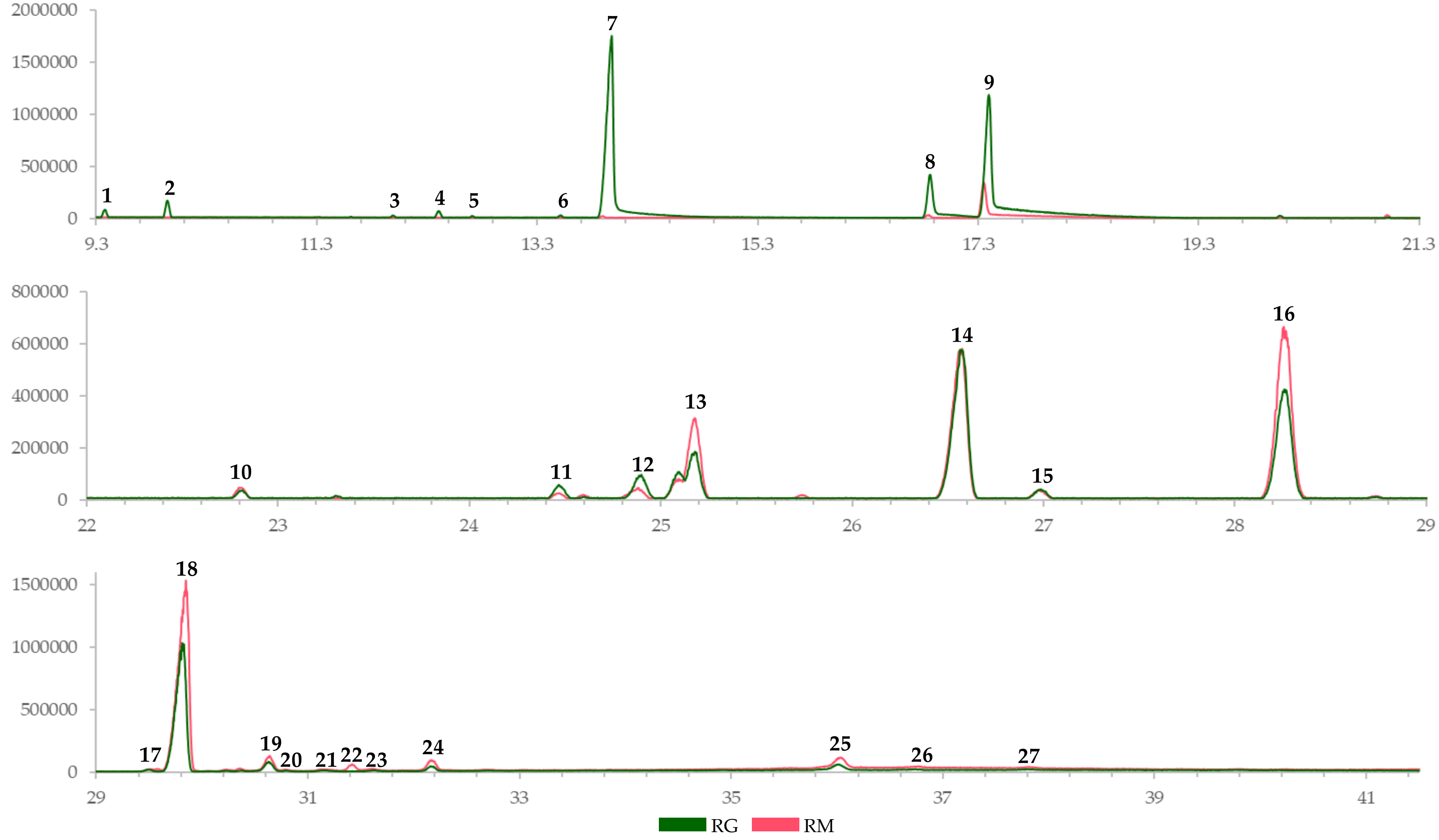

Figure S8. Overlaid and zoomed-in base peak chromatograms (BPC) of *Piper rubro-venosum* leaf essential oils in GC-MS analysis I. Constituents of numbered peaks are listed in **Table S3**.

Table S4. Constituents of *Piper rubro-venosum* leaf essential oils identified in GC-MS analysis IIa*. Constituent peaks are labelled in **Figure S9**.

No.	RT (min)	LRI	Constituent	Reference
1	10.12	1000	δ-2-Carene	[35]
2	10.25	1005	*m*-Cymene	[37]
3	10.39	1010	*p*-Mentha-1(7),8-diene	[38]
4	10.49	1014	Eucalyptol	[39]
5	10.75	1024	(*Z*)-β-Ocimene	[35]
**6**	**11.05**	**1035**	**(*E*)-β-Ocimene**	[35]
**7**	**11.41**	**1049**	**γ-Terpinene**	[36]
**8**	**11.82**	**1064**	**(*Z*)-Sabinene hydrate**	[36]
**9**	**12.43**	**1087**	**Terpinolene**	[35]
10	12.51	1090	Linalool oxide A	[35]
**11**	**12.94**	**1105**	**Linalool**	[35]
12	13.74	1127	(*Z*)-*p*-Menth-2-en-1-ol	[35]
13	14.37	1146	(*E*)-*p*-Menth-2-en-1-ol	[35]
14	15.35	1174	δ-Terpineol	[35]
**15**	**15.66**	**1182**	**Terpinen-4-ol**	[35]
**16**	**16.3**	**1201**	**α-Terpineol**	[35]
17	16.53	1207	(*Z*)-Piperitol	[35]
18	16.87	1218	(*E*)-Piperitol	[35]
19	17.31	1230	(*E*)-Chrysanthenyl acetate	[36]
20	17.58	1238	Nerol	[40]
21	18.29	1259	Linalool acetate	[36]
22	18.61	1269	Geraniol	[35]
**23**	**21.01**	**1339**	**δ-Elemene**	[35]
24	21.42	1350	α-Cubebene	[35]
25	21.98	1367	Cyclosativene	[36]
26	22.19	1372	α-Ylangene	[36]
**27**	**22.36**	**1378**	**α-Copaene**	[35]

No.	RT (min)	LRI	Constituent	Reference
**35**	**24.2**	**1424**	**(*E*)-β-Caryophyllene**	[35]
**36**	**24.46**	**1430**	**β-Copaene**	[35]
37	24.69	1436	γ-Elemene	[36]
38	24.84	1439	Aromadendrene	[36]
39	25.08	1444	Isogermacrene D	[41]
**40**	**25.57**	**1456**	**α-Humulene**	[35]
41	25.65	1457	Sesquisabinene	[36]
**42**	**26.43**	**1475**	**γ-Muurolene**	[36]
**43**	**27.05**	**1489**	**Germacrene D**	[35]
44	27.14	1492	Eremophilene	[42]
45	27.33	1496	γ-Amorphene	[36]
46	27.59	1502	4-*epi*-Cubebol	[35]
**47**	**27.73**	**1504**	**α-Muurolene**	[35]
**48**	**27.99**	**1509**	**Germacrene A**	[35]
**49**	**28.25**	**1513**	**β-Curcumene**	[35]
**50**	**28.37**	**1515**	**γ-Cadinene**	[35]
51	28.65	1521	Cubebol	[35]
**52**	**28.88**	**1525**	**δ-Cadinene**	[35]
53	29.3	1532	10-*epi*-Cubebol	[36]
54	29.56	1537	α-Cadinene	[35]
55	30.06	1546	(*Z*)-Sesquisabinene hydrate	[36]
56	30.4	1552	α-Elemol	[43]
57	30.58	1555	7-*epi*-(*E*)-Sesquisabinene hydrate	[44]
58	31.86	1578	Germacrene D-4-ol	[35]
59	32	1581	Spathulenol	[35]
**60**	**32.23**	**1585**	**Caryophyllene oxide**	[35]
61	32.8	1595	(*E*)-Sesquisabinene hydrate	[35]

Table S4 (continued)

No.	RT (min)	LRI	Constituent	Reference	No.	RT (min)	LRI	Constituent	Reference
**28**	**22.7**	**1388**	**β-Elemene**	[36]	**62**	**33.77**	**1610**	**Humulene epoxide II**	[35]
29	22.9	1393	β-Cubebene	[35]	63	34.95	1628	Epicubenol	[36]
30	23.02	1397	Isocaryophyllene	[35]	64	35.88	1642	*epi*-α-Muurolol	[35]
31	23.12	1400	β-Longipinene	[36]	65	36.19	1647	α-Muurolol	[35]
32	23.37	1405	(*Z*)-α-Bergamotene	[35]	66	36.81	1656	α-Cadinol	[35]
33	23.53	1409	7-*epi*-Sesquithujene	[38]	67	38.87	1686	4(15),5,10(14)-Germacratrien-1-ol	[36]
34	23.75	1414	α-Cedrene	[35]	68	68.48	2122	Phytol	[35]

Note: Constituents in bold were identified in GC-MS analysis I and IIa.

*Parameter settings were as in GC-MS II, except: Solvent delay time = 10 min, scan rate = 10 scans/s, m/z range = 40-700. Oven temperature settings = 50 °C (1.0 min), 4 °C/min to 130 °C (0.0 min), 1 °C/min to 170 °C (0.0 min), 5 °C/min to 240 °C (5.0 min). Total run time = 80 min. Minimum integrated area > 0.01% of the largest peak in the TIC chromatograms.

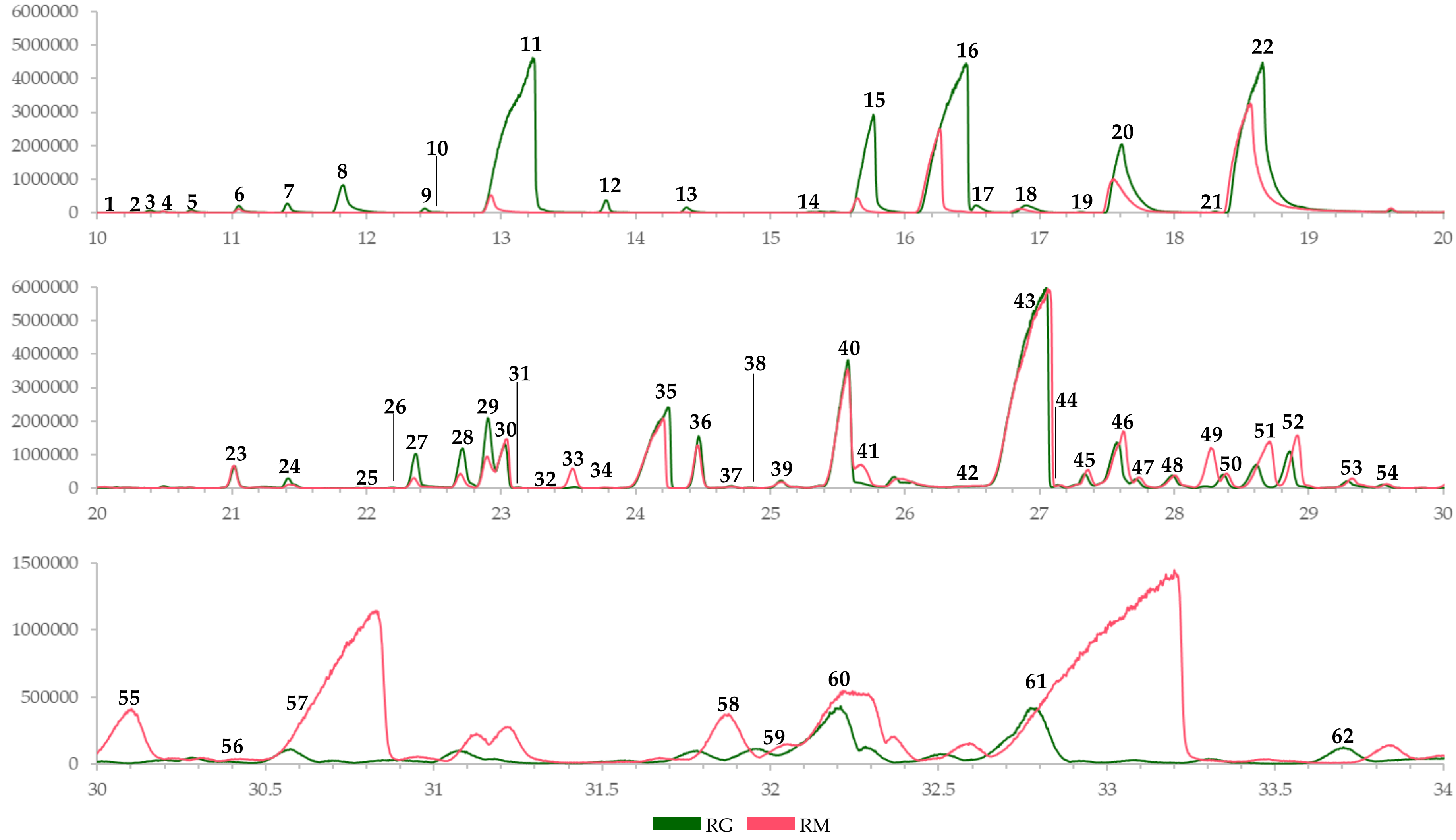

Figure S9. Overlaid and zoomed-in base peak chromatograms (BPC) of *Piper rubro-venosum* leaf essential oils in GC-MS analysis IIa. Constituents of numbered peaks are listed in **Table S4**.

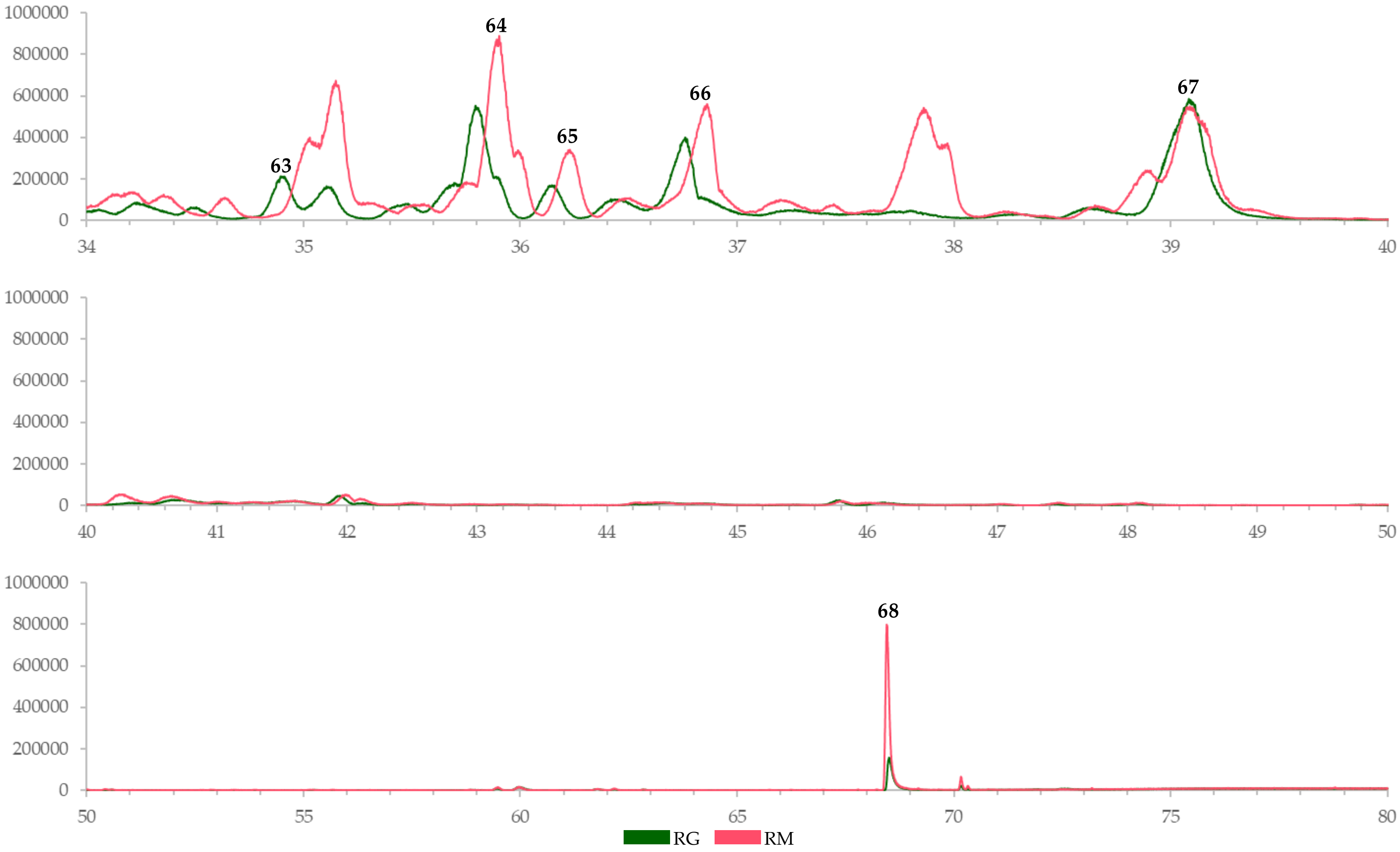

Figure S9 (continued)

Table S5. Constituents of *Piper betle* leaf essential oils identified in GC-MS analysis I. Constituent peaks are labelled in **Figure S10**.

No.	RT (min)	LRI	Constituent	Reference
**1**	**11.41**	**1031**	**Eucalyptol**	[35]
**2**	**11.63**	**1037**	**(*Z*)-β-Ocimene**	[35]
**3**	**12.01**	**1048**	**(*E*)-β-Ocimene**	[35]
**4**	**13.92**	**1100**	**Linalool**	[35]
**5**	**17.49**	**1196**	**Methyl salicylate**	[35]
6	17.61	1199	Methyl chavicol	[36]
**7**	**22.82**	**1338**	**δ-Elemene**	[35]
**8**	**23.10**	**1346**	**Chavicol acetate**	[45]
**9**	**24.26**	**1373**	**Chavibetol**	[46]
**10**	**25.14**	**1394**	**β-Elemene**	[35]
**11**	**25.59**	**1404**	**Methyl eugenol**	[35]
**12**	**26.25**	**1417**	**(*Z*)-α-Bergamotene**	[35]
**13**	**26.56**	**1423**	**(*E*)-β-Caryophyllene**	[35]
**14**	**28.26**	**1455**	**α-Humulene**	[35]
**15**	**29.49**	**1478**	**γ-Muurolene**	[35]
**16**	**29.78**	**1484**	**Germacrene D**	[35]
**17**	**30.06**	**1489**	**β-Selinene**	[35]
**18**	**30.64**	**1500**	**Bicyclogermacrene**	[35]
**19**	**31.23**	**1509**	**(*E*,*E*)-α-Farnesene**	[35]
**20**	**31.64**	**1515**	**γ-Cadinene**	[35]
**21**	**32.19**	**1524**	**δ-Cadinene**	[35]
**22**	**32.71**	**1532**	**Chavibetol acetate**	[36]
**23**	**34.35**	**1557**	**Germacrene B**	[36]
**24**	**40.72**	**1647**	**4-Allyl-1,2-diacetoxybenzene**	[47]

Note: Constituents in bold were identified in GC-MS analysis I and II.

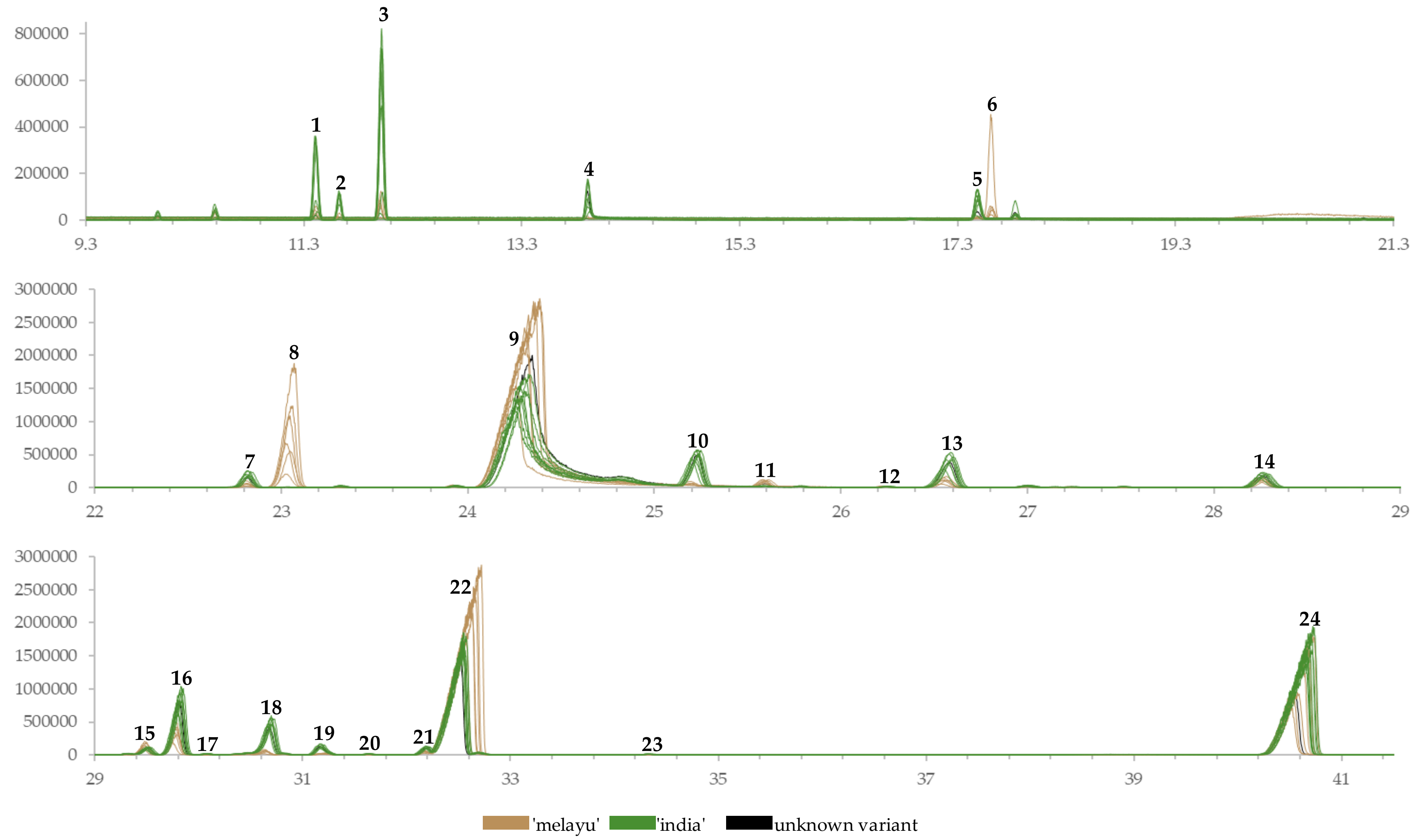

Figure S10. Overlaid and zoomed-in base peak chromatograms (BPC) of *Piper betle* leaf essential oils in GC-MS analysis I. Constituents of numbered peaks are listed in **Table S5**.

Table S6. Constituents of *Piper betle* leaf essential oils identified in GC-MS analysis II. Constituent peaks are labelled in **Figure S11**.

No.	RT (min)	LRI	Constituent	Reference
0	6.12	900	*n*-Nonane (Internal standard)	-
1	7.51	947	Camphene	[36]
2	8.35	976	β-Pinene	[36]
3	8.80	991	Myrcene	[36]
4	10.02	1029	Limonene	[35]
**5**	**10.11**	**1031**	**Eucalyptol**	[35]
**6**	**10.34**	**1038**	**(*Z*)-β-Ocimene**	[35]
**7**	**10.69**	**1049**	**(*E*)-β-Ocimene**	[35]
**8**	**12.54**	**1103**	**Linalool**	[35]
9	15.23	1181	Terpinen-4-ol	[35]
10	15.73	1196	α-Terpineol	[35]
**11**	**15.83**	**1199**	**Methyl salicylate**	[35]
12	18.45	1280	(*E*)-Anethole	[35]
**13**	**20.55**	**1336**	**δ-Elemene**	[36]
**14**	**20.99**	**1348**	**Chavicol acetate**	[45]
15	21.51	1362	Eugenol	[35]
16	21.81	1371	α-Copaene	[36]
**17**	**21.95**	**1375**	**Chavibetol**	[46]
18	22.56	1392	(*Z*)-Isoeugenol	[48]
**19**	**22.64**	**1394**	**β-Elemene**	[36]
**20**	**23.33**	**1410**	**Methyl eugenol**	[35]
**21**	**23.57**	**1415**	**(*Z*)-α-Bergamotene**	[36]
**22**	**23.81**	**1420**	**(*E*)-Caryophyllene**	[36]
23	24.16	1427	β-Copaene	[36]
24	24.35	1432	γ-Elemene	[36]
25	24.58	1436	Aromadendrene	[36]
26	25.10	1447	(*Z*)-Muurola-3,5-diene	[36]

Note: Constituents in bold were identified in GC-MS analysis I and II.

No.	RT (min)	LRI	Constituent	Reference
**27**	**25.26**	**1451**	**α-Humulene**	[36]
28	25.42	1454	(*E*)-β-Farnesene	[36]
29	25.56	1457	Alloaromadendrene	[35]
**30**	**26.43**	**1476**	**γ-Muurolene**	[36]
**31**	**26.66**	**1480**	**Germacrene D**	[36]
**32**	**26.88**	**1485**	**β-Selinene**	[36]
33	27.11	1490	(*Z*)-β Guaiene	[36]
**34**	**27.45**	**1497**	**Bicyclogermacrene**	[36]
35	27.58	1500	α-Muurolene	[36]
36	27.85	1505	α-Bulnesene	[36]
**37**	**28.03**	**1508**	**(*E*,*E*)-α-Farnesene**	[36]
**38**	**28.29**	**1512**	**γ-Cadinene**	[36]
**39**	**28.81**	**1521**	**δ-Cadinene**	[36]
40	29.26	1529	(*E*)-γ-Bisabolene	[36]
**41**	**29.50**	**1533**	**Chavibetol acetate**	[36]
**42**	**30.60**	**1552**	**Germacrene B**	[35]
43	31.33	1564	Maaliol	[36]
44	32.09	1577	Spathulenol	[36]
45	32.28	1580	Caryophyllene oxide	[36]
46	32.40	1582	Gleenol	[35]
47	32.89	1591	Viridiflorol	[36]
48	33.55	1602	Rosifoliol	[36]
49	34.47	1615	Junenol	[36]
50	35.21	1625	Epicubenol	[36]
51	35.44	1629	Eremoligenol	[36]
52	36.54	1644	α-Muurolol	[36]
**53**	**36.90**	**1649**	**4-Allyl-1,2-diacetoxybenzene**	[47]

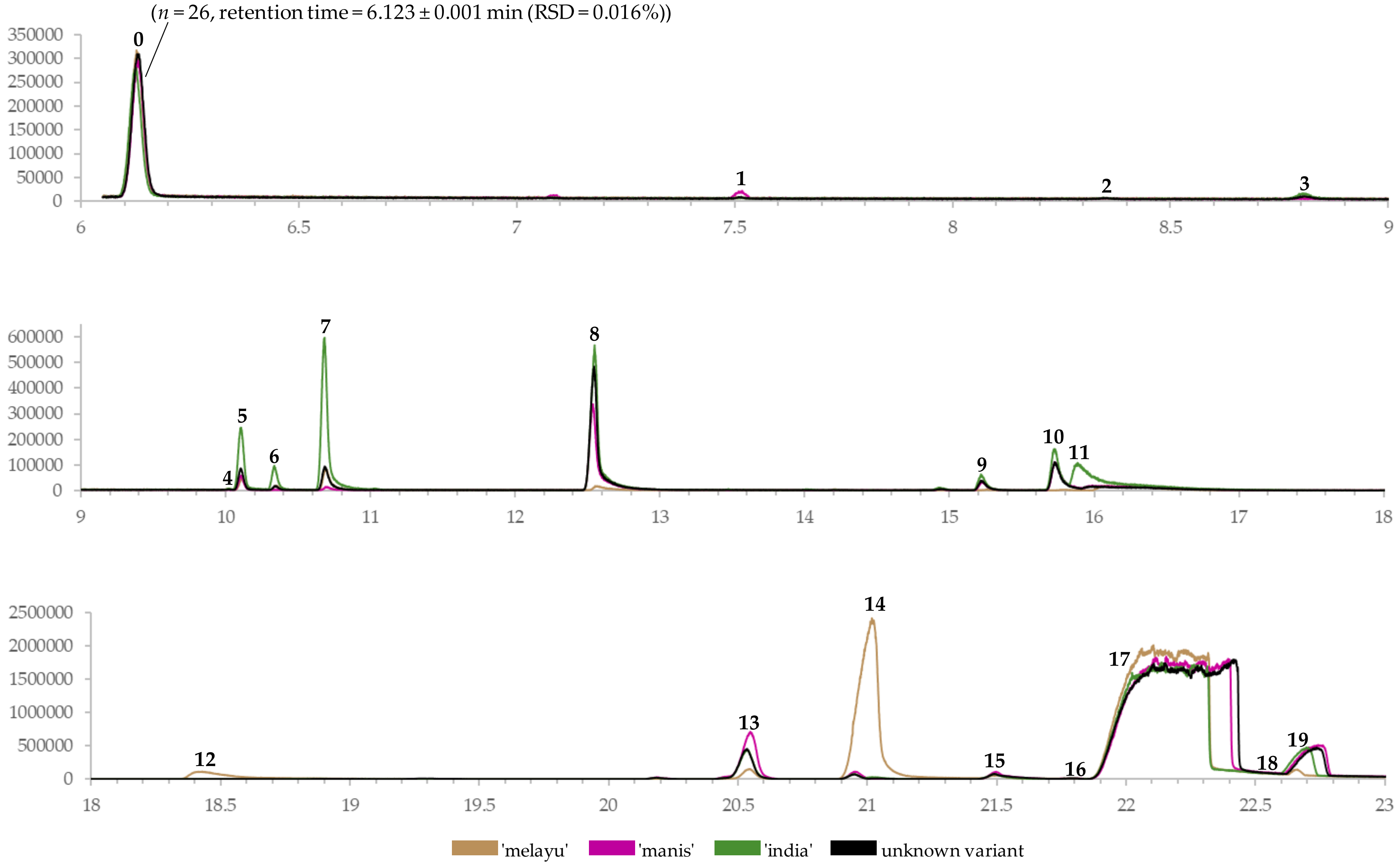

Figure S11. Representative BPC of *Piper betle* leaf essential oils in GC-MS analysis II. Constituents of numbered peaks are listed in **Table S6**.

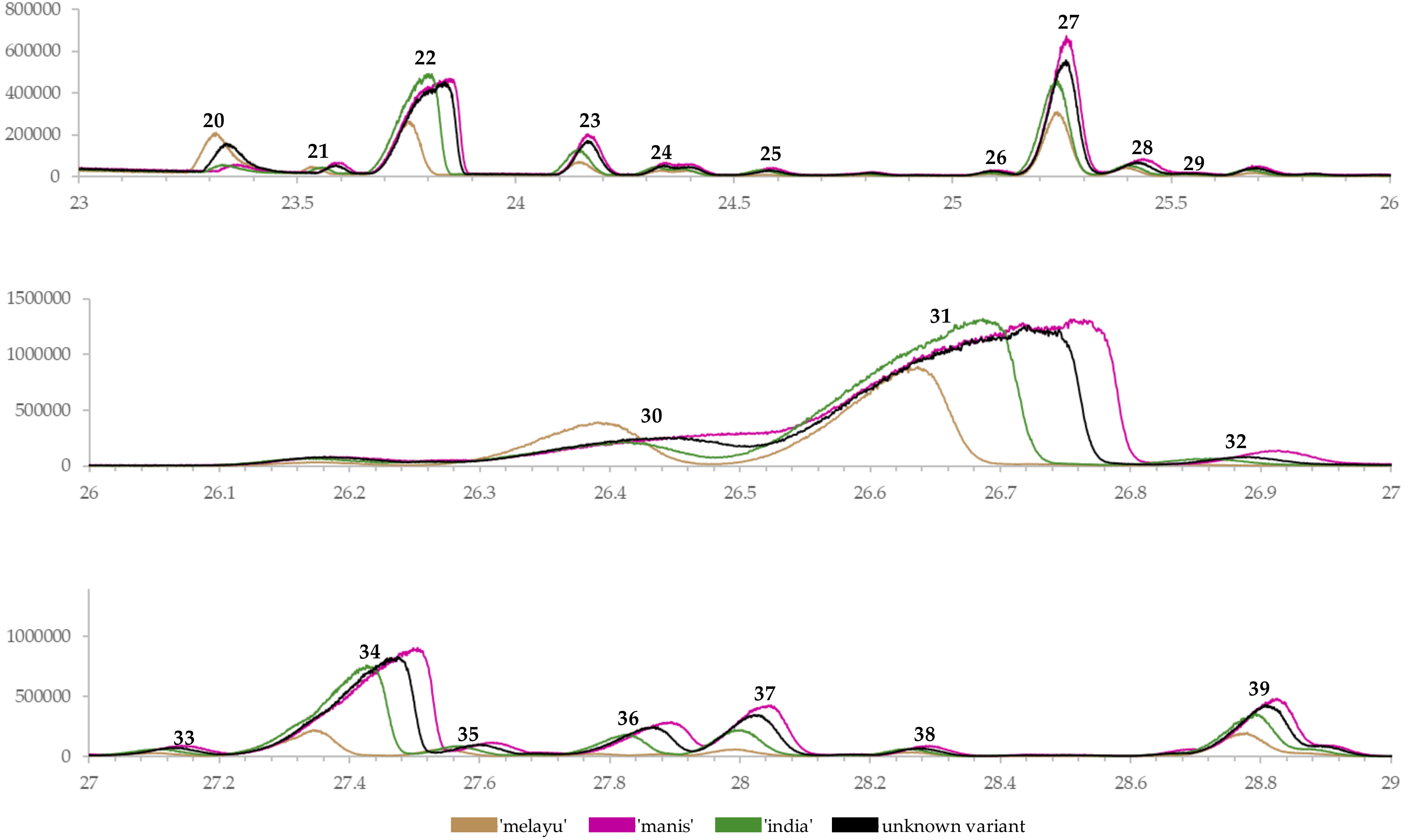

Figure S11 (continued)

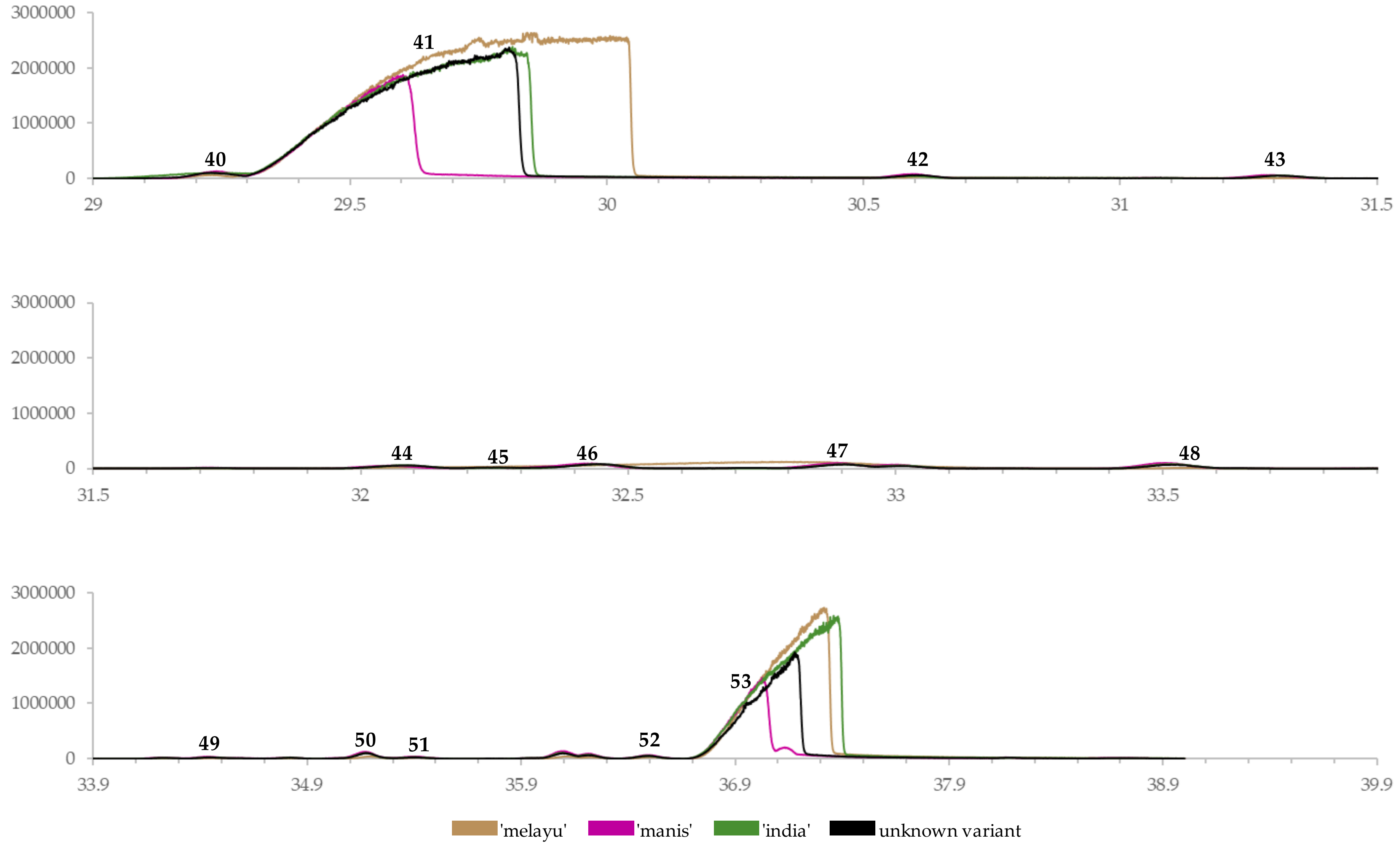

Figure S11 (continued)

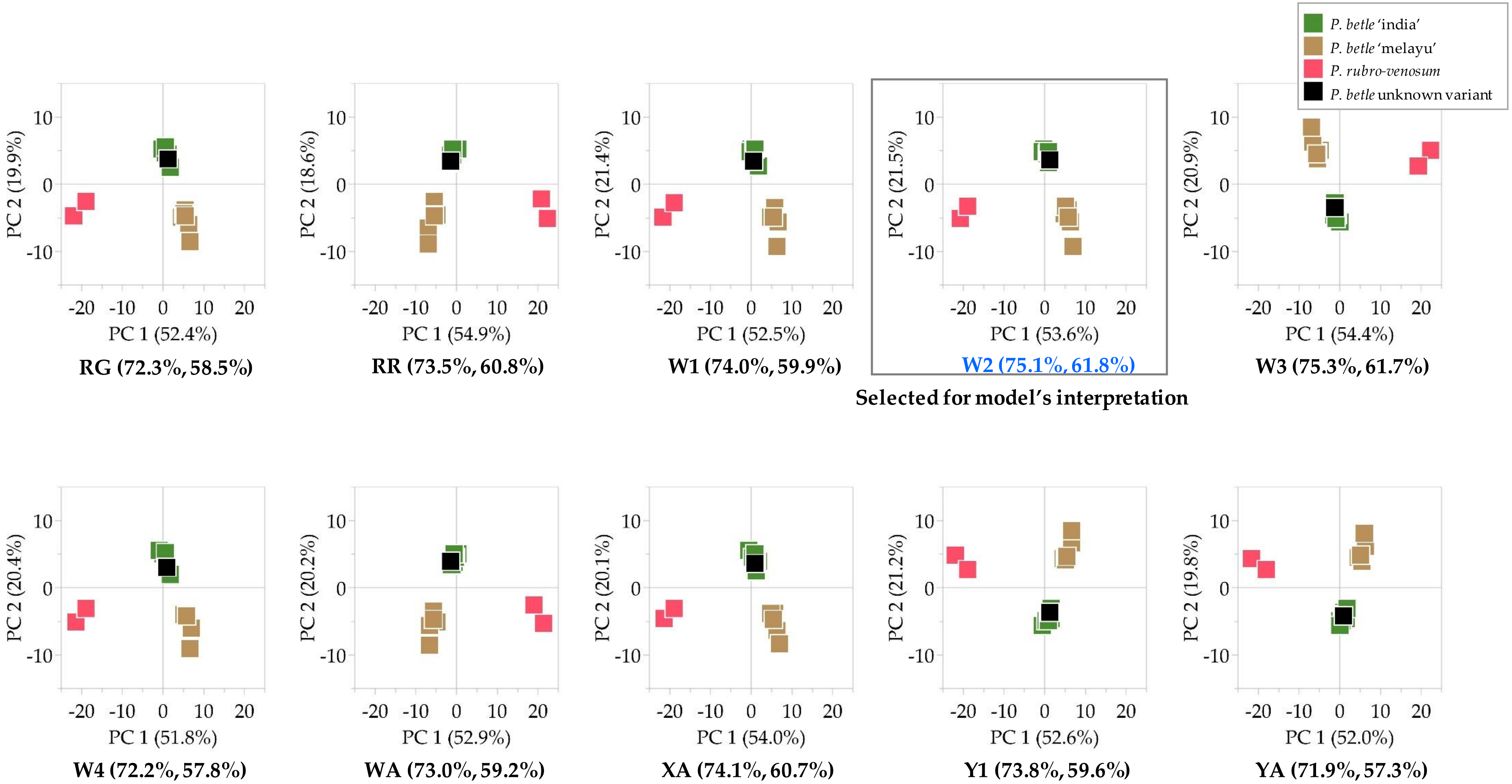

Figure S12. Score plots of principal component (PC) 1 *versus* 2 for GC-MS analysis I data when different reference files were used in MS-DIAL. The reference file, R^2X (cumulative up to PC 2), and Q^2X (cumulative up to PC 2) are highlighted in bold.

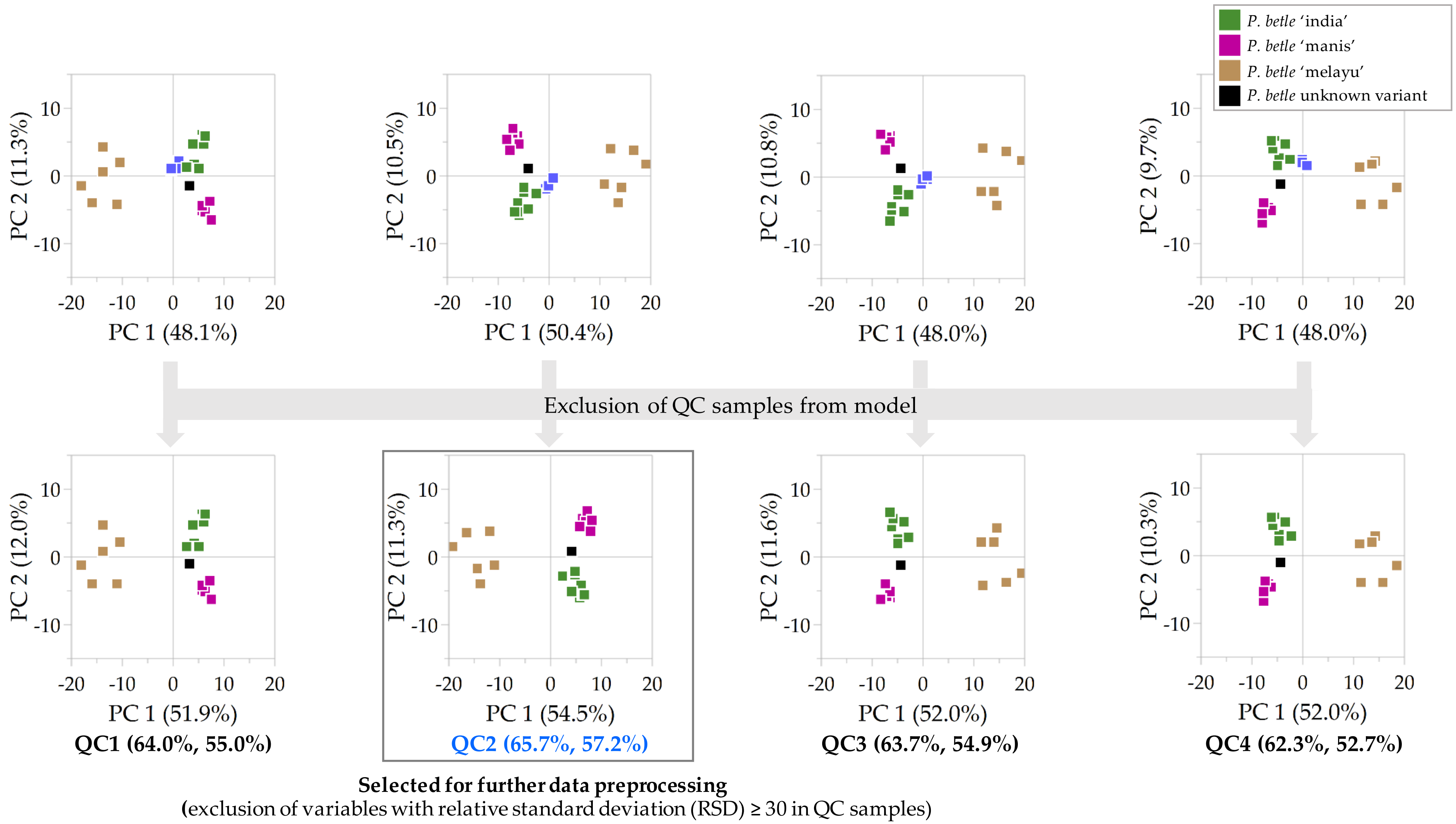

Figure S13. Score plots of principal component (PC) 1 *versus* 2 for GC-MS analysis II data when different reference files were used in MS-DIAL. The reference file, R^2X (cumulative up to PC 2), and Q^2X (cumulative up to PC 2) are highlighted in bold.

Table S7. Parameter settings in MS-DIAL version 4.24.

GC-MS	I	II
**Data type**		
Data type	Centroid	
Ion mode	Positive	
Accuracy type	Nominal	
**Data collection parameters**		
Retention time begin	9.30	6.05
Retention time end	41.50	39.00
Mass range begin	39.60	40.00
Mass range end	250.00	249.80
**Data processing**		
Number of threads	8	
**Peak detection parameters**		
Smoothing method	Linear weighted moving average	
Smoothing level	3	
Average peak width	20	
Minimum peak height	1000	
Mass slice width	0.5	
Mass accuracy	0.5	
**MS1 Deconvolution parameters**		
Sigma window value	0.5	
Amplitude cut off	2000	

GC-MS	I	II
**Alignment parameters setting**		
Reference file	W2	QC2
Retention time	RI	
Retention index tolerance	10	
Retention time tolerance	0.075	
EI similarity tolerance	80	
Retention time factor	0.5	
EI similarity factor	0.5	
Identification after alignment	FALSE	
Gap filling by compulsion	FALSE	
Base peak m/z selected as the representative quant mass	FALSE	
**Filtering setting**		
Peak count filter	5	
Remove feature based on peak height fold-change	FALSE	
Sample max / blank average	5	
Sample average / blank average	5	
Keep identified and annotated metabolites	FALSE	
Keep removable features and assign the tag for checking	TRUE	
**Export option**		
Raw data matrix (Height)	FALSE	
Normalized data matrix	TRUE	
Filtering by the ion abundances of blank samples	TRUE	

References

35. Babushok, V.I.; Linstrom, P.J.; Zenkevich, I.G. Retention indices for frequently reported compounds of plant essential oils. *J. Phys. Chem. Ref. Data* **2011**, *40*, 1–47. https://doi.org/10.1063/1.3653552
36. Adams, R.P. *Identification of Essential Oil Components by Gas Chromatography/Mass Spectrometry*, 4.1 ed.; Allured Publishing: Illinois, USA, 2017.
37. García, D.; Alvarez, A.; Tornos, P.; Fernandez, A.; Sáenz, T. Gas chromatographic-mass spectrometry study of the essential oils of *Pimenta racemosa* var. *terebinthina* and *P. racemosa* var. *grisea*. *Z. Naturforsch., C, J. Biosci.* **2002**, *57*, 449–451. https://doi.org/10.1515/znc-2002-5-608
38. Cardeal, Z.L.; Gomes da Silva, M.D.R.; Marriott, P.J. Comprehensive two-dimensional gas chromatography/mass spectrometric analysis of pepper volatiles. *Rapid Commun. Mass Spectrom.* **2006**, *20*, 2823–2836. https://doi.org/10.1002/rcm.2665
39. Yassaa, N.; Meklati, B.Y.; Cecinato, A. Evaluation of monoterpenic biogenic volatile organic compounds in ambient air around *Eucalyptus globulus*, *Pinus halepensis* and *Cedrus atlantica* trees growing in Algiers city area by chiral and achiral capillary gas chromatography. *Atmos. Environ.* **2000**, *34*, 2809–2816. https://doi.org/10.1016/S1352-2310(99)00436-7
40. Perez-Cacho, P.R.; Mahattanatawee, K.; Smoot, J.M.; Rouseff, R. Identification of sulfur volatiles in canned orange juices lacking orange flavor. *J. Agric. Food Chem*. **2007**, *55*, 5761–5767. https://doi.org/10.1021/jf0703856
41. Radulović, N.; Lazarević, J.; Ristić, N.; Palić, R. Chemotaxonomic significance of the volatiles in the genus *Stachys* (Lamiaceae): Essential oil composition of four Balkan *Stachys* species. *Biochem. Syst. Ecol.* **2007**, *35*, 196–208. https://doi.org/10.1016/j.bse.2006.10.010
42. Bortolomeazzi, R.; Berno, P.; Pizzale, L.; Conte, L.S. Sesquiterpene, alkene, and alkane hydrocarbons in virgin olive oils of different varieties and geographical origins. *J. Agric. Food Chem*. **2001**, *49*, 3278–3283. https://doi.org/10.1021/jf001271w
43. Lo Presti, M.; Sciarrone, D.; Crupi, M.L.; Costa, R.; Ragusa, S.; Dugo, G.; Mondello, L. Evaluation of the volatile and chiral composition in *Pistacia lentiscus* L. essential oil. *Flavour Fragr. J.* **2008**, *23*, 249–257. https://doi.org/10.1002/ffj.1878
44. Weyerstahl, P.; Marschall, H.; Thefeld, K.; Subba, G.C. Constituents of the essential oil from the rhizomes of *Hedychium gardnerianum* Roscoe. *Flavour Fragr. J.* **1998**, *13*, 377–388. https://doi.org/10.1002/ffj.2730100310
45. NIST Chemistry WebBook, SRD 69. Available online: https://rb.gy/zy2yw9 (accessed on 20 August 2019).
46. dos Santos, B.C.B.; da Silva, J.C.T.; Guerrero, P.G.; Leitão, G.G.; Barata, L.E.S. Isolation of chavibetol from essential oil of *Pimenta pseudocaryophyllus* leaf by high-speed counter-current chromatography. *J. Chromatogr. A* **2009**, *1216*, 4303–4306. https://doi.org/10.1016/j.chroma.2009.01.111
47. Mohottalage, S.; Tabacchi, R.; Guerin, P.M. Components from Sri Lankan *Piper betle* L. leaf oil and their analogues showing toxicity against the housefly, *Musca domestica*. *Flavour Fragr. J.* **2007**, *22*, 130–138. https://doi.org/10.1002/ffj.1770
48. Saroglou, V.; Dorizas, N.; Kypriotakis, Z.; Skaltsa, H.D. Analysis of the essential oil composition of eight *Anthemis* species from Greece. *J. Chromatogr. A* **2006**, 1104, 313–322. https://doi.org/10.1016/j.chroma.2005.11.087
